# Predictors of Helmet CPAP Failure in COVID-19 Pneumonia: A Prospective, Multicenter, and Observational Cohort Study

**DOI:** 10.1155/2022/1499690

**Published:** 2022-01-21

**Authors:** Pierachille Santus, Stefano Pini, Francesco Amati, Marina Saad, Marina Gatti, Michele Mondoni, Francesco Tursi, Maurizio Rizzi, Davide Alberto Chiumello, Valter Monzani, Francesco Blasi, Stefano Aliberti, Dejan Radovanovic

**Affiliations:** ^1^Division of Respiratory Diseases, Ospedale Luigi Sacco, Polo Universitario, ASST Fatebenefratelli-Sacco, Milano, Italy; ^2^Department of Biomedical and Clinical Sciences (DIBIC), Università degli Studi di Milano, Milano, Italy; ^3^Respiratory Unit, IRCCS Humanitas Research Hospital, Rozzano, Italy; ^4^Respiratory Unit, ASST Santi Paolo e Carlo, San Paolo Hospital, Milano, Italy; ^5^Dipartimento di Scienze Della Salute, Università degli Studi di Milano, Milano, Italy; ^6^UOS Pneumologia, Ospedale Civico di Codogno, ASST Lodi, Lodi, Italy; ^7^Department of Anesthesia and Intensive Care, ASST Santi Paolo e Carlo, San Paolo University Hospital, Milano, Italy; ^8^Coordinated Research Center on Respiratory Failure, Università Degli Studi di Milano, Milano, Italy; ^9^Fondazione IRCCS Ca' Granda Ospedale Maggiore Policlinico, Department of Medicine, Acute Medical Unit, Milano, Italy; ^10^Fondazione IRCCS Ca' Granda Ospedale Maggiore Policlinico, Respiratory Unit and Cystic Fibrosis Adult Center, Milano, Italy; ^11^Università Degli Studi di Milano, Department of Pathophysiology and Transplantation, Milano, Italy; ^12^Department of Biomedical Sciences, Humanitas University, Pieve Emanuele, Milano, Italy

## Abstract

**Background:**

Continuous positive airway pressure (CPAP) can be beneficial in acute respiratory failure (ARF) due to coronavirus (COVID-19) pneumonia, but delaying endotracheal intubation (ETI) in nonresponders may increase mortality. We aimed at investigating the performance of composite respiratory indexes as possible predictors of CPAP failure in ARF due to COVID-19.

**Methods:**

This was a multicenter, prospective, observational, and cohort study conducted in the respiratory units of three University hospitals in Milan and in a secondary care hospital in Codogno (Italy), on consecutive adult patients with ARF due to COVID-19 pneumonia that underwent CPAP between March 2020 and March 2021. ETI transfer to the intensive care unit or death is defined CPAP failure. Predictors of CPAP failure were assessed before T0 and 1 hour after T1 CPAP initiation and included mROX index (ratio of PaO2/FiO2 to respiratory rate), alveolar-to-arterial (A-a) O_2_ gradient, and the HACOR (heart rate, acidosis, consciousness, oxygenation, and respiratory rate) score.

**Results:**

Three hundred and fifty four patients (mean age 64 years, 73% males) were included in the study; 136 (38.4%) satisfied criteria for CPAP failure. A-a O_2_ gradient, mROX, and HACOR scores were worse in patients who failed CPAP, both at T0 and T1 (*p* < 0.001 for all parameters). The HACOR score was associated with CPAP failure (odds ratio—OR—for every unit increase in HACOR = 1.361; 95%CI: 1.103–1.680; *p*=0.004; AUROC = 0.742; *p* < 0.001). CPAP failure was best predicted by a threshold of 4.50 (sensitivity = 53% and specificity = 87%).

**Conclusions:**

The HACOR score may be a reliable and early predictor of CPAP failure in patients treated for ARF in COVID-19 pneumonia.

## 1. Introduction

Continuous positive airway pressure (CPAP) is a valuable noninvasive respiratory support to treat acute hypoxic respiratory failure (hypoxic ARF) associated with coronavirus (COVID-19) pneumonia [1–3]. CPAP may avoid unnecessary endotracheal intubation (ETI) [4]; however, delaying invasive ventilation in nonresponders to CPAP may increase mortality [4]. Early and reliable predictors of CPAP failure in COVID-19 pneumonia are still lacking [1, 5, 6].

Our aim was to investigate and compare the performance of different composite respiratory indexes in identifying CPAP failure in patients affected by COVID-19 pneumonia and ARF.

## 2. Materials and Methods

This was a multicenter, prospective, observational, and cohort study conducted in the high dependency respiratory units (HDRU) of three academic hospitals in Milan (Luigi Sacco University Hospital, Ospedale Maggiore Policlinico, and San Paolo University Hospital) and one secondary care hospital in Codogno (Italy). Consecutive adult patients with ARF due to laboratory confirmed COVID-19 pneumonia that underwent CPAP between March 2020 and March 2021 were enrolled. Exclusion criteria were ETI or death <24 hours from hospital admission.

CPAP was started by protocol as previously reported [1] and was delivered through high-flow generators (VitalSigns Inc; 90–140 L·min^−1^; MYO 3133A, Pulmodyne) using a helmet (StarMed) with an adjustable PEEP valve (VitalSigns) [1]. Anthropometrical characteristics, vital signs, and blood gas analysis were collected before CPAP (T0) and 1 hour after CPAP positioning (T1). FiO_2_ before CPAP was estimated by administration of oxygen via Venturi masks. Blood tests and chest X-ray were performed at T0. Hospital length of stay and all-cause in-hospital mortality were registered. According to local standard operating procedures, ceiling treatment and eligibility for ETI were judged by the treating physician and the critical care staff. Criteria for ETI and a “do-not-intubate” (DNI) order were previously reported [1]. In case of CPAP failure, noninvasive ventilation (NIV) could be instituted in the ICU based on clinical judgement and bed availability.

CPAP failure was defined as the need for ETI and/or transfer to the ICU or death in HDRU in DNI patients. Predictors of CPAP failure were assessed at T0 and T1 and included (1) mROX index (ratio of partial pressure of oxygen to inspired oxygen fraction and respiratory rate: PaO_2_/FiO_2_/RR) [7], (2) alveolar-to-arterial (A-a) O_2_ gradient, and (3) HACOR score, a composite index (0–25 points, the higher the worse) in which clinical items are scored and stratified based on severity, including heart rate (0–1), respiratory acidosis (0–4), and level of consciousness (Glasgow Coma Scale–GCS, 0–10), PaO_2_/FiO_2_ (0–6), and RR (0–4) [8].

The study (NCT04307459) followed the amended Declaration of Helsinki (2013), which was approved by the local ethical committees (No. 17263/2020, No. 345/2020, and No. 2020/ST/095), and all patients gave written informed consent.

Analyses were performed with IBM SPSS Statistics for Windows, V.23.0 (Armonk, NY). Variables were expressed as median and interquartile range (IQR) or means and standard deviation (SD), according to their distribution assessed with the Shapiro–Wilk test. Independent groups *T*-test, Chi-square, or Mann–Whitney tests were used to compare CPAP successes and failures. A logistic regression analysis was performed to investigate determinants of CPAP failure. The area under the receiver operating characteristic curve (AUROC) was calculated for CPAP failure predictors at T0 and T1. Tests were two sided and statistical significance was taken at *p* < 0.05.

## 3. Results

Three hundred and fifty four patients (mean age 64 years, 73% males) were included in the study, of which 136 (38.4%) satisfied criteria for CPAP failure (Table 1). Fifty-one (14.4%) patients received a DNI order, of which 32 (62.7%) died. Ninety seven (27.4%) patients were transferred to the ICU, of which 64 (65.8%) were intubated. Seven (7.2%) patients failed CPAP and received invasive mechanical ventilation while in the HDRU (Table 1). In-hospital mortality was 27.1% while ICU mortality was 66%. Among patients who were not directly intubated in the ICU, 20.6% received NIV and all eventually undergone ETI. Mortality in patients exposed to NIV did not significantly differ compared with patients intubated after CPAP failure (60.0% vs. 58.4%, *p*=0.742).

Patients that failed CPAP were older, more likely to have chronic comorbidities, and had worse clinical severity, hypoxemia, A-a O_2_ gradient, mROX, and HACOR scores at T0 than patients who succeeded it, respectively, 326 (96–604) mmHg vs. 179 (56–315) mmHg, 4.3 (2.3–6.5) mmHg/bpm vs. 7.3 (4.8–11.1) mmHg/bpm, and 6 (3.0–6.3) vs. 2 (0–5.0) (Table 1). After CPAP initiation, respiratory parameters tended to improve in both groups, but were still deteriorated in patients that failed CPAP (Table 1).

**Figure 1:**
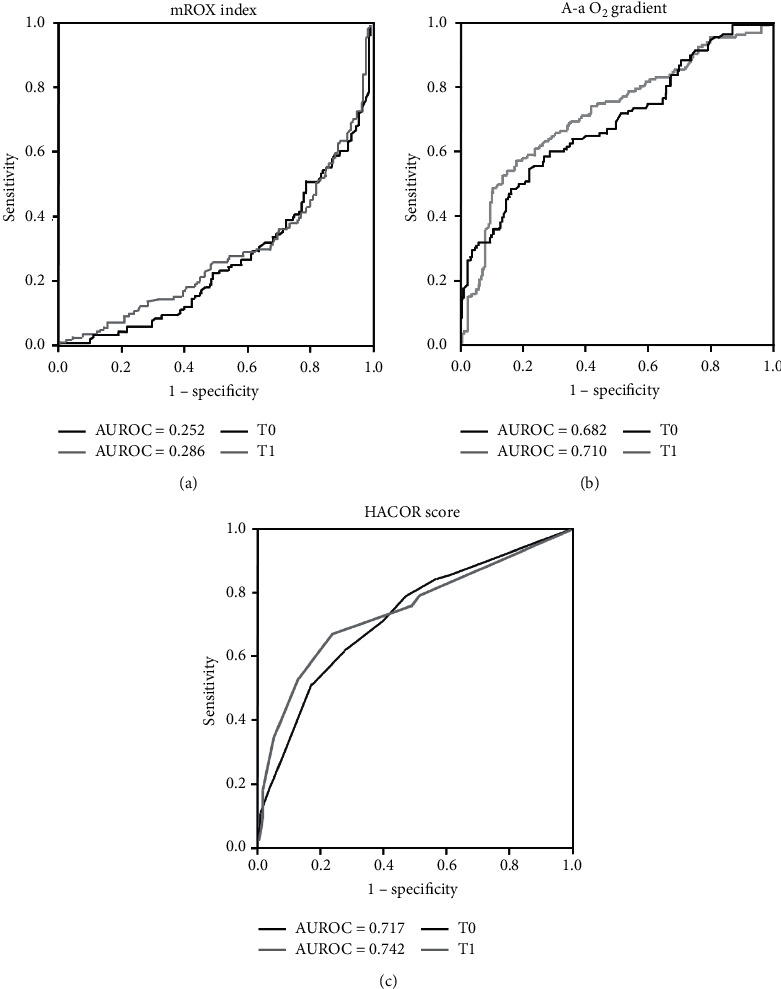
Accuracy of predictors of CPAP failure. Receiver operating characteristic (ROC) curves with areas under the ROC curves showing the performance for mROX index (a), A-a O_2_ gradient (b), and HACOR score (c) in predicting CPAP failure at T0 (black lines) and T1 (grey lines). mROX index = modified ROX index (PaO_2_/FiO_2_/respiratory rate); A-a O_2_ gradient = alveolar-arterial oxygen gradient; HACOR = heart rate, acidosis (pH), consciousness (Glasgow coma scale: GCS), oxygenation, and respiratory rate score; AUROC = area under the receiver operating characteristic curve.

The logistic regression analysis, including age, history of ischemic heart disease and chronic pulmonary disease, D-dimer and C-reactive protein at admission, PaO_2_/FiO_2_, RR, mROX, A-a O_2_ gradient, and HACOR score at T1 as independent variables, showed that the only parameter significantly associated with CPAP failure was the HACOR score (odds ratio—OR—for every unit increase in HACOR = 1.361; 95%CI:1.103–1.680; *p*=0.004; AUROC = 0.742; *p* < 0.001) (Figure 1). The threshold that best discriminated CPAP failure was 4.50 (sensitivity, i.e., true positive rate = 53%; specificity, i.e., true negative rate = 87%). The results did not change excluding DNI patients or after performing a sensitivity analysis that sequentially removed data relative to each participating center (data not shown).

## 4. Discussion

To date, only a limited number of studies explored predictors for CPAP failure in patients with COVID-19 pneumonia, with inconsistent results. CPAP failure was shown to be independently predicted by pneumonia severity and interleukin-6 level at admission [1], by the presence of hypertension [5], or, more recently, by a combination of age, gender, lactic dehydrogenase, and change in PaO_2_/FiO_2_ after CPAP positioning (relative risk 95%CI: 0.998, 0.996–0.999) [6].

The mROX index, an evolution of the ROX index (ratio of the percentage of arterial oxygen saturation, measured via a pulse oximeter, and respiratory rate: SaO_2_/FiO_2_/RR) [9], is a predictor of failure of high-flow nasal cannula (HFNC) in patients with ARF [7]. The ROX index, but not the mROX index, has been recently investigated as a potential predictor of HFNC failure in patients with COVID-19 pneumonia [10].

The HACOR score is a validated predictor of failure of noninvasive ventilation (NIV) and HFNC, both in acute respiratory failure [8, 11] and in respiratory failure in patients with COPD [12].

In patients with ARF, CPAP improves both hypoxemia and work of breathing [2]. In COVID-19 pneumonia, however, the application of CPAP has shown to improve oxygenation to a variable extent, with unclear effects on patients' outcomes [1, 5, 13, 14], suggesting that the *a priori* estimation of CPAP efficacy judged solely on the severity of hypoxia may lead to an underestimation of CPAP potential positive effects [15–17].

The HACOR score could help to evaluate response to CPAP because it is a composite index that includes surrogates of gas exchange efficiency (PaO_2_/FiO_2_), clinical severity (heart rate, GCS), and respiratory distress (RR, pH), thus overcoming the limits of using just a potentially unreliable index like PaO_2_/FiO_2_ [15]. The mROX also combines a noninvasive surrogate of respiratory distress (RR) and gas exchange efficiency (PaO_2_/FiO_2_). The mROX, as a difference from the ROX index, uses PaO_2_ instead of SaO_2_ to estimate the oxygenation in patients with ARF; this parameter requires an arterial blood gas analysis (ABG) to be performed, thus is more invasive than just relying on pulse oximeter, but avoids the intrinsic inaccuracies related to measuring and interpreting SaO_2_ [15].

We also observed that patients that failed CPAP had persistently elevated A-a O_2_ gradient and respiratory rate despite the ventilatory support, sustaining the hypothesis that severe ventilation/perfusion mismatch and consequent profound hypoxemia may cause exhaustion and lead to ETI. In this view, Corradi et al. have recently conducted a study that suggested that the diaphragmatic performance, as assessed by ultrasonography, which may be an accurate predictor of CPAP failure in patients with ARF due to COVID-19 [18].

Our results suggest that HACOR score predicts early CPAP failure in COVID-19 patients with high specificity, which could be of high clinical interest for resource allocation and monitoring optimization. Interestingly, patients that failed CPAP had a median HACOR score of 5, which corresponds to the threshold that predicted NIV failure in the original validation study [8]. Instead, the limited sensitivity could be ascribed to the low number of patients having positive scores in the “pH” and “GCS” items in our population.

The severity of respiratory failure and the rate of CPAP failure were comparable with previous studies [1, 5, 6], and CPAP failure was not influenced by timing of initiation or by in-hospital treatment. Helmet NIV has been shown to be superior to HFNC in avoiding ETI in patients with severe COVID-19 pneumonia [19]. Moreover, recent evidence suggested that a noninvasive support escalation strategy based on a NIV trial after CPAP failure might reduce mortality and avoid unnecessary ETI, while not deteriorating patients' clinical status or delaying intubation [20].

The present study has limitations. The scores should be tested at different time points, to assess the variability of their performance after CPAP positioning. Changes in patients' severity and healthcare system preparedness during the observation year may have influenced the clinical outcomes [21]. Finally, the combined primary end point of the study may have biased the accuracy of predictive indexes (e.g., mortality in DNI patients). The sensitivity analyses, however, sustained the robustness of the results. Further studies are needed to prospectively validate the score in COVID-19 patients.

## 5. Conclusions

In conclusion, the HACOR score may be a reliable and early bedside predictor of CPAP failure in patients with ARF due to COVID-19 and could help identifying patients at higher risk of unfavorable outcome and that might benefit from an early targeted pharmacological intervention, a prompt escalation, to noninvasive ventilation or from the institution of invasive mechanical ventilation.

## Figures and Tables

**Table 1:** Clinical characteristics at admission and after CPAP positioning and predictors of CPAP failure

Characteristics (*n* = 354)	All CPAP patients (*n* = 354)	CPAP success (*n* = 218)	CPAP failure (*n* = 136)	*p* value^a^
Age (years)	64 (56–73)	62 (54–71)	69 (60–76)	<0.001
Males, *n* (%)	260 (73.4)	164 (75.2)	96 (70.6)	0.336
Hypertension, *n* (%)	164 (46.3)	98 (45)	66 (48.5)	0.512
Diabetes mellitus, *n* (%)	80 (22.6)	45 (20.6)	35 (25.7)	0.275
Ischemic heart disease, *n* (%)	56 (15.8)	21 (9.6)	35 (25.7)	<0.001
COPD, *n* (%)	25 (7.1)	10 (4.6)	15 (11)	0.021
DNI, *n* (%)	51 (14.4)	19 (8.7)	32 (23.5)	<0.001
In-hospital treatment (*n* = 272)				
Systemic corticosteroids, *n* (%)	190 (69.9)	107 (69.0)	83 (70.9)	0.734
Tocilizumab, *n* (%)	45 (12.7)	25 (11.5)	20 (9.2)	0.682
LMWH, prophylactic, *n* (%)	103 (37.9)	67 (43.2)	36 (30.8)	0.067
LMWH, therapeutic, *n* (%)	156 (57.4)	84 (54.2)	72 (61.5)	0.084
Outcomes (*n* = 354)				
Time to CPAP start, days	2 (1–4)	2 (1–4)	1 (0–3)	0.086
CPAP duration, days	6 (3–9)	6 (4–10)	4 (3–8)	<0.001
Hospital length of stay, days	16 (11–25)	18 (13–26)	11 (6–20)	<0.001
Admitted to ICU, *n* (%)	97 (27.4)	0	97 (71.3)	N.A.
Received NIV after CPAP failure in ICU, *n* (%)	20 (20.6)	0	20 (20.6)	N.A.
ETI, *n* (%)	71 (20.1)	0	71 (52.2)	N.A.
ETI after NIV, *n* (%)	20 (100)	0	20 (100)	N.A.
ETI not moved to ICU, *n* (%)	7 (2.0)	0	7 (5.1)	N.A.
In-hospital mortality, *n* (%)	96 (27.1)	0	96 (70.6)	N.A.
ICU mortality, *n* (%)	64 (66.0)^¶^	0	65 (66.0)^¶^	N.A.
Mortality after positioning NIV, *n* (%)	12 (60.0)	0	12 (60.0)	N.A.
Mortality among DNI, *n* (%)	32 (9.0)	0	32 (23.5)	N.A.
Clinical variables at admission				
White blood cells (×10^6^/L)	7.37 (5.50–10.11)	7.06 (5.4–9.6)	8.5 (5.7–11.6)	0.019
Platelets (×10^6^/L)	214 (165–298)	221 (172–303)	201 (148–284)	0.052
D-dimer (*µ*g/L FEU)	854 (489–1425)	709 (425–1199)	1084 (642–2456)	<0.001
CRP (mg/L)	34 (12–114)	31 (11–92)	39 (15–143)	0.039
Body temperature (°C)	37.1 (36.4–38)	37 (36.4–37.8)	37.4 (36.5–38)	0.067
CPAP parameters				
PEEP (cmH_2_O)	10 (7.5–10)	10 (7.5–10)	10 (8–12)	0.002
FiO_2_ (%)	60 (50–60)	60 (50–60)	60 (60–70)	<0.001
Respiratory and clinical parameters before CPAP positioning (T0)				
pH	7.47 (7.45–7.50)	7.47 (7.45–7.50)	7.48 (7.45–7.51)	0.808
PaCO_2_ (mmHg)	33 (29–37)	33 (30–37)	32 (29–35)	0.058
PaO_2_/FiO_2_ (mmHg)	161 (100–252)	194 (122–273)	110 (68–181)	<0.001
A-a O_2_ gradient (mmHg)	239 (61–432)	179 (56–315)	326 (96–604)	<0.001
Respiratory rate (bpm)	27 (23–30)	25 (22–30)	28 (24–34)	<0.001
Glasgow coma scale^*∗*^	15 (15–15)	15 (15–15)	15 (15–15)	0.001
ROX index (%/bpm)	8 (4.4–14.8)	9.5 (5.9–15.9)	5.4 (3.3–11.1)	<0.001
mROX index (mmHg/bpm)	6.1 (3.6–9.7)	7.3 (4.8–11.1)	4.3 (2.3–6.5)	<0.001
HACOR score	4.0 (0–6.0)	2 (0–5.0)	6 (3.0–6.3)	<0.001
Respiratory and clinical parameters 1 hour after CPAP positioning (T1)				
pH	7.46 (7.44–7.49)	7.46 (7.44–7.49)	7.47 (7.43–7.49)	0.812
PaCO_2_ (mmHg)	36 (33–40)	37 (34–40)	35 (31–39)	0.004
PaO_2_/FiO_2_ (mmHg)	188 (126–251)	207 (161–265)	132 (108–220)	<0.001
A-a O_2_ gradient (mmHg)	254 (196–308)	230 (183–279)	302.6 (234–330)	<0.001
Respiratory rate (bpm)	24 (20–28)	24 (20–28)	26 (22–30)	<0.001
Glasgow coma scale^*∗∗*^	15 (15–15)	15 (15–15)	15 (15–15)	0.011
ROX index (%/bpm)	6.9 (5.6–8.5)	7.6 (6.2–9.2)	6.2 (4.7–7.7)	<0.001
mROX index (mmHg/bpm)	7.6 (5.1–10.9)	8.8 (6.7–12.1)	5.8 (3.8–8.9)	<0.001
HACOR score	2 (0–5)	1 (0–3)	5 (2–6)	<0.001

Data are presented as medians (interquartile range), unless otherwise stated. DNI, “do-not-intubate” order; A-a O_2_ gradient, alveolar-arterial oxygen gradient; bpm, breaths per minute; CI, confidence interval; CPAP, continuous positive airway pressure; CRP, C-reactive protein (upper limit of normal = 10 mg/L); FEU, fibrinogen equivalent units; GCS, Glasgow coma scale; PaO_2,_ arterial partial pressure of oxygen; PaCO_2,_ arterial partial pressure of carbon dioxide; ROX index, SpO_2_/FiO_2_/respiratory rate; mROX index, modified ROX index (PaO_2_/FiO_2_/respiratory rate); HACOR, heart rate, acidosis (pH), consciousness (Glasgow coma scale: GCS), oxygenation, and respiratory rate; AUROC, area under the receiver operating characteristic curve. ^a^Comparison between CPAP success and CPAP failure. ^*∗*^denotes seven (3.2%) patients in the success group vs. 16 (11.8%) patients in the failure group had a GSC <15. ^*∗∗*^ denotes eight (3.7%) patients in the success group vs. 15 (11%) patients in the failure group had a GSC <15. ^¶^denotes among patients admitted to ICU.

## Data Availability

The data used to support the findings of the study are available from the corresponding author upon request.
